# 

**Published:** 1867-03

**Authors:** 


### SUBSCRIBERS

Who have ordered other publications through this office, will please address them directly, in case of failure to receive the papers to which they are entitled. The press of new subscribers has been such that back numbers can not be always obtained, but the subscriptions will run into next year to make up. Business men will make allowance for the delays incident to the extra work of the first of the year, as also for those errors in transcription which are inseparable from the large amount of labor in other offices, where a thousand subscriptions come in daily for weeks together, requiring, sometimes, successive new editions to be printed. It will be a saving of time, trouble, and money if, in writing to publishers, persons would simply write on the reverse side of an envelope which has been addressed to them by some one else, cutting off the part used in sealing up. Some of the advantages are: It would save half a sheet of good paper; it would prevent writing a great deal, thus saving time, trouble, and patience to the publisher in trying to decipher hieroglyphics, for few persons are able, or do, write their names with sufficient distinctness to be easily and quickly deciphered by a total stranger, whereas almost any man will write another's name plainly; and from having received the envelope through the post-office, it would contain name, town, and State, all in full, which millions of writers do not give in their letters, even, as the dead-letter post-office shows, when large amounts of money are contained. Publishers receive a good many dollars in letters without any name or place being written within.

All subscriptions to Hall's Journal of Health must begin with the January number; each number averages twenty pages of reading matter, and is not promised to be mailed before the first day of the month for which it is issued. Orders for the Journal of Health are filled the very hour they are received.

